# Comparing nodal versus bony metastatic spread using tumour phylogenies

**DOI:** 10.1038/srep33918

**Published:** 2016-09-22

**Authors:** Stefano Mangiola, Matthew K. H. Hong, Marek Cmero, Natalie Kurganovs, Andrew Ryan, Anthony J. Costello, Niall M. Corcoran, Geoff Macintyre, Christopher M. Hovens

**Affiliations:** 1 Departments of Urology and Surgery, Royal Melbourne Hospital and University of Melbourne, Parkville 3050 Victoria, Australia; 2 Centre for Neural Engineering, 203 Bouverie St, Carlton 3053, Victoria, Australia; 3 TissuPath Specialist Pathology, Mount Waverley 3149, Victoria, Australia; 4 The Epworth Prostate Centre, Epworth Hospital, Richmond 3121, Victoria, Australia; 5 Cancer Research UK Cambridge Institute, University of Cambridge, Cambridge CB2 0RE, UK

## Abstract

The role of lymph node metastases in distant prostate cancer dissemination and lethality is ill defined. Patients with metastases restricted to lymph nodes have a better prognosis than those with distant metastatic spread, suggesting the possibility of distinct aetiologies. To explore this, we traced patterns of cancer dissemination using tumour phylogenies inferred from genome-wide copy-number profiling of 48 samples across 3 patients with lymph node metastatic disease and 3 patients with osseous metastatic disease. Our results show that metastatic cells in regional lymph nodes originate from evolutionary advanced extraprostatic tumour cells rather than less advanced central tumour cell populations. In contrast, osseous metastases do not exhibit such a constrained developmental lineage, arising from either intra or extraprostatic tumour cell populations, at early and late stages in the evolution of the primary. Collectively, this comparison suggests that lymph node metastases may not be an intermediate developmental step for distant osseous metastases, but rather represent a distinct metastatic lineage.

It has long been recognized that the spread and seeding of metastatic tumour cells from the primary site of origin does not follow a random pattern, rather, there are characteristic anatomical sites favouring the formation of metastatic lesions^1^. For epithelial tumours such as prostate adenocarcinoma, the bony skeleton as well as regional lymph nodes are a common site of metastatic spread. Depending on the tumour stage and grade, as well as the anatomical extent of dissection, lymph node metastases can be identified in up to 8% of patients undergoing radical prostatectomy in contemporary series^2^,^3^,^4^. However the clinical behaviour of lymph node metastases differs from that of bony and other visceral disease. Patients presenting with lymph node only metastases have significantly better progression-free and overall survival compared to patients with mixed lymph node and bony disease, bony disease alone, or spread to other visceral sites, and consistently demonstrate an improved therapeutic response to hormonal manipulation and chemotherapy^5^. In addition, in a randomised study evaluating the impact of early androgen deprivation in patients with N1 disease, 14% of patients were disease free at a median of 11.9 years postoperatively with no further treatment, suggesting at least the possibility of long term disease control if not cure with surgical excision alone^6^.

For over a century, lymph nodes have been considered to have an essential role in the metastasis of epithelial tumours^7^, acting as a staging ground for wider visceral dissemination^8^. Recent clinical studies however suggest that metastatic spread is a more complex process, and that tumour colonisation of regional lymph nodes may not directly contribute to lethality, but instead represent an evolutionary ‘cul-de-sac’ that may be a pathological marker of inherently aggressive disease but does not play a direct role in systemic colonisation^9^. Importantly it remains to be clarified whether visceral metastases arise from previously established metastatic lymph nodes or if lymph nodes are irrelevant to the evolution of distant metastases. The understanding of the role of lymph node metastases in prostate cancer evolution will provide key insights into the mechanisms leading to the lethal disease.

Recent studies in prostate cancer^10^,^11^ used tumour phylogenies to reveal the complexity of distant tumour spread, describing patterns of cross metastatic seeding, multiple metastatic waves of spread from the primary, and a possible metastatic origin of local recurrences.

Several evolutionary models that attempt to describe the dynamic processes driving tumour cell diversification and spread to distant organs have been proposed in the pre-next-generation sequencing era, including clonal evolution^12^ the mutator phenotype^12^,^13^ and stochastic progression^14^. Using next-generation sequencing data, algorithms that reconstruct phylogenies of metastatic cancers based on nucleotide variants have been recently developed^15^. Tumour phylogenetic methods that permit the tracing of tumour cell lineages in human cancers have been used to identify the roots of metastasis in humans^16^ and, along with mapping in a comprehensive manner the sub-clones within the primary tumour, has been stressed to be key for the reconstruction of the metastatic spread^17^. However, these studies did not characterise localised lymph node metastases.

To address this, we collected a total of 19 samples from multiple prostate tumour foci from 3 patients, together with matched metastatic lymph nodes. We performed genome-wide single nucleotide polymorphism (SNP) array profiling of each sample and characterised all copy-number changes. We then combined these data with 26 samples from 3 men with distant metastatic disease^11^. In order to identify the patterns of copy number aberrations (CNAs) that emerged at the transition across histo-pathological categories, a combined evolutionary reconstruction and differential analysis of gene copy number was performed. Using these copy-number profiles we were able to derive tumour phylogenetic trees for each patient using MEDICC^18^, combined with a bootstrapping procedure to refine and improve robustness of the trees. Each tree was then validated using targeted Multiplex Ligation-dependent Probe Amplification (MLPA) of 58 genomic regions spanning 39 genes. Analysis of the tumour phylogenies revealed distinct patterns of metastatic spread between the two patient groups, as well as potential drivers of transition across histo-pathological categories. We show lymph node metastases originated exclusively from evolutionarily advanced, extraprostatic regions of primary tumour, whereas distant metastases originated either from central (2 cases) or extraprostatic primary tumour regions (1 case).

## Results

### A comparison of broad copy-number changes between tumour locations identifies shared and unique events

**Figure 1:**
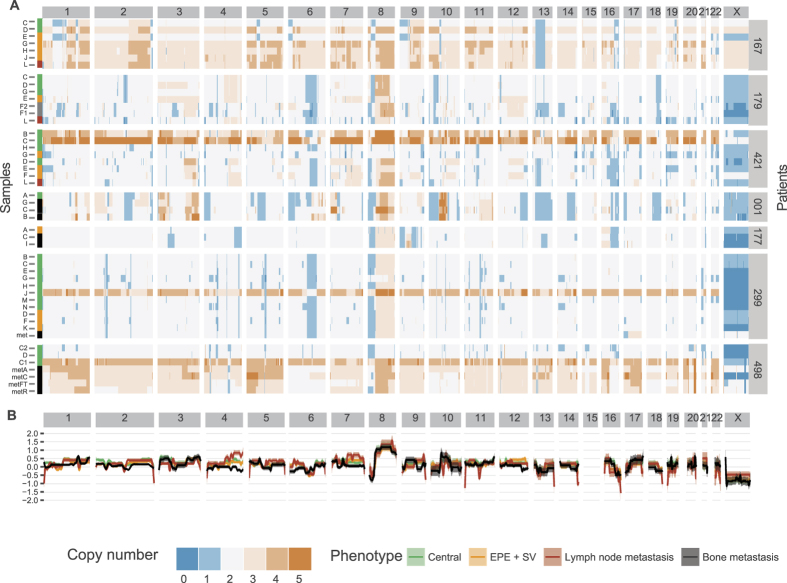
(**A**) Heat map of gene copy numbers across chromosomes. The copy number profiles were consistently fragmented across samples to allow comparative analyses. (**B**) Loess regression of the CNA profiles of the sampled clones adjusted for total ploidy. The value of 0 of the y axes represents no increase/decrease compared to a diploid genome.

We used the Illumina BeadChip to determine the genome-wide copy-number profiles of 48 samples collected from 6 patients with metastatic prostate cancer (see Methods section). 37 were inferred as approximately diploid, 10 were found with to have an average ploidy close to 3, and 1 sample had a tetraploid genome (Fig. 1A, Table S1). Primary and metastatic disease sites were found to have examples of both diploid and non-diploid genomes.

We grouped the samples by histo-pathological category (central, extraprostatic, lymph node, and bony metastasis), adjusted for ploidy, and computed a summary copy-number profile (Fig. 1B). Visual inspection of the profiles indicated shared changes across the groups: recurrent 8p deletions (8p22 to 8p11.23) and 8q amplifications (8q11.21 to 8q24.23), were observed similar to previous studies^19^, along with deletion of the long arm of chromosome 16 (16q22.1 to 16q24.3)^20^. Given these changes are shared across groups, they are likely to have occurred multiple times through evolution of each tumour.

The only region to show marked divergent frequencies between groups was 4q (Fig. 1B), with primary tumour samples showing no change, lymph node metastases showing amplification and bony metastases showing deletion. This region harbours TET2, previously shown to be deleted in metastatic disease^21^ and its expression linked with disease progression^22^.

### Tumour phylogenies reveal the origin of lymph node metastases as exclusively extraprostatic

**Figure 2:**
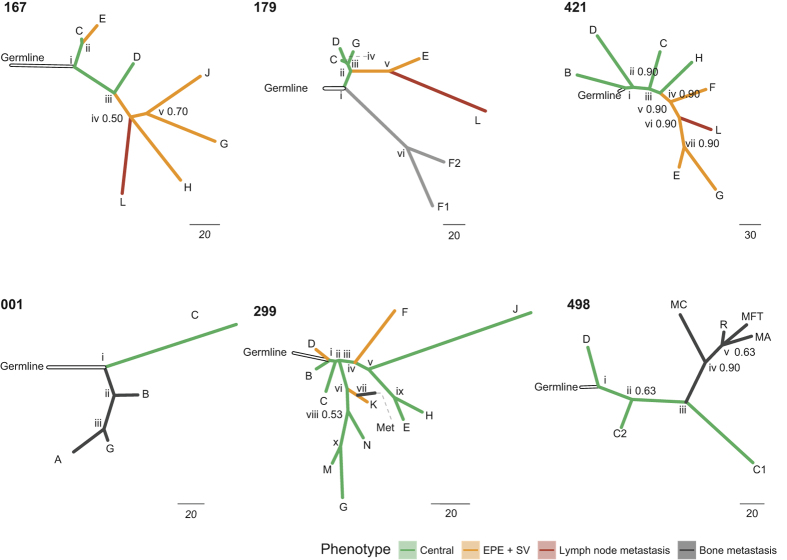
Cancer evolutionary hierarchies for patients 167, 179, 421 (including lymph node metastasis) and 001, 299, 498 (including distant metastasis). The roman numbers indicate the predicted ancestral cell populations. The decimal numbers at branches represent the confidence values of the clade prediction (when smaller then 1).

In order to identify clones and infer their evolutionary hierarchy in each patient we used MEDDIC^18^ in combination with a bootstrapping strategy to improve tree robustness (see Methods section). The resulting phylogenies (Fig. 2) show that lymph node metastases have a recurrent evolutionary pattern originating exclusively from regions of extraprostatic extension (EPE), never directly from clones present within the central prostatic capsule. In contrast, the distant osseous metastatic clones originated from either central or extraprostatic locations. Central origin can be seen in patients 001 and 498 where no extraprostatic, localized tumour was identified upon pathological review, either in the SV or EPE. In these two cases all of the distant metastases (i.e., C, B and G for patient 001; and MC, R, MFT and MA for patient 498) originated from an ancestor (i.e., ii and iii respectively) derived from a tumour locus localized in a deep, central position in the prostate suggesting an early tumour population within the primary cancer. In contrast, for patient 299, the distant, bony shoulder metastasis (i.e., Met) was derived from a tumour population located extraprostatically in the seminal vesicle (i.e., K)^11^.

**Figure 3:**
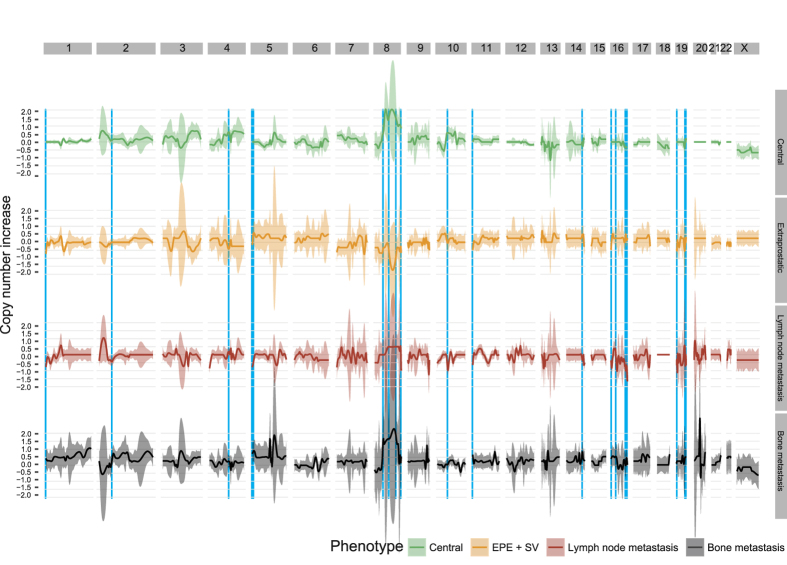
Loess regression of the increase/decrease CNA profiles, obtained from the difference in copy numbers across transition between predicted ancestral representative of the four histo-pathological categories (i.e., from diploid-to-central tumour, central-to-EPE, EPE-to-lymph node metastasis, and central/EPE-to-bony metastasis) for each patient (one per patient, per category). The value of 0 of the y axes represents no variation compared to the parent histo-pathological category. The light-blue shades represent the genomic regions having a differential amount of copies across the four transitions (p-value <0.1, after method Benjamini, Hochberg, and Yekutieli multiple test correction^44^).

In order to validate these observations we used an orthogonal copy-number profiling approach, Multiplex Ligation-dependent Probe Amplification (MLPA) technology. We processed samples C, D, G, H, J and L from patient 167; C, D, E, G and L from patient 179; and C, E, F, G, H and L from patient 421. For the selected samples we tested a total of 39 genes, utilizing a total of 63 different probes. Overall, we validated the direction of variation in copy number (i.e., deletion, amplification or diploid) for over 71% of the probes across all selected samples. For each branch of the evolutionary hierarchies, we validated an average of 68% of the probes for the genes that define that branching (Table 1). Although MLPA technology was not designed for taking into account samples with highly variable tumour purity, we could obtain a validation p-value <10^−4^ for each branch along the evolutionary schema (Table 1), using a binomial test (binom.test function in R; using 0.33 for the p parameter, indicating three possible categorical outcomes for copy number changes: decrease, increase, no-change). The evolutionary hierarchies for patients 001, 177, 299 and 498 were consistent with previous analyses based on whole genome sequencing data^11^. For patient 001 (Fig. 3), both our analyses and published data indicate that the distant metastases had a common central tumour ancestor, namely, tumour sample C. Similarly for patient 498, the distant metastases consistently clustered together having a single common ancestor derived from a central primary tumour. For patient 299, the relationship between the various metastatic and primary tumour foci for this patient was derived from the WGS data^11^, due to biases in the SNP array data as described.

### Tumour phylogenies facilitate identification of copy-number changes associated with metastatic progression

We used the inferred clonal phylogenies to associate specific copy-number events with the transition from diploid-to-central tumour, central-to-EPE, EPE-to-lymph node metastasis, and central/EPE-to-bony metastasis (see Methods, Fig. 3, and Table 2).

A number of interesting observations can be made from this analysis including refinements of the broad copy-number changes observed earlier. The analysis shows that 8p deletions are observed at low frequencies across all four transitions, albeit at slightly higher frequencies in the transition to bony metastases, suggesting a potential necessary condition for spread to bone. Broad 8q gains appear at high frequency during the emergence of the central tumour, however, in the transition to EPE, there is a focal deletion (within the base pair interval 77796946–116276618). This likely represents a loss-of-heterozygosity that confers ability to spread and invade (Fig. 3). Further amplification of 8q is observed in both transitions to lymph and bone metastases, likely as a result of the necessity to increase c-Myc gene copy-number, an important regulator of proliferation^23^.

16q deletions were detected as significantly altered, at low frequency in central tumour development and metastasis to bone, and at high frequency in the development of lymph node metastases. This region includes FBXO31, a key oncosuppressor in prostate cancer that commonly displays loss of heterozygosity, including in breast, ovarian and hepatocellular cancers^24^. This alteration may be necessary for the ability to metastasise from the EPE to lymph nodes. Two other loci in chromosome 16 also show differential behaviour between categories (Table 2): 16p13.3 from chromosome 16, which contains CBP, the transcriptional co-activator of CREB (cAMP response element binding protein); this gene regulates cell differentiation, proliferation and survival and is involved in prostate tumour initiation^25^. Additionally, the locus 16q12 from chromosome 16 had a decrease in copy number during the transition to lymph node. This region includes two key genes: CYLD and BRD7. The gene CYLD is a deubiquitination enzyme which regulates cell survival or cell proliferation, and is commonly lost in different types of human cancer^26^; the gene BRD7 negatively regulates cell proliferation and growth and has a key role in prostate cancer development^27^.

Further alterations can be found in 11p15.5 from chromosome 11, which is decreased in both the transition to extraprostatic tumour and to lymph node metastases. This region includes MUC2, a known tumour suppressor^28^; and MUC5, which have been previously described as under-expressed in prostate cancer^28^.

The gene TET2 located in the locus 4q24 increased in copy number with higher frequency in the transition to central tumour and lower frequencies in the transition to lymph node and bony metastases. Finally, three other loci showed altered copy numbers: 4q24, 5p15.2 and 19q13.4, containing potential drivers in NPNT, SEMA5A and BRSK1 respectively.

### Global copy-number profile comparison identifies lymph and bony metastases as distinct disease states

**Figure 4:**
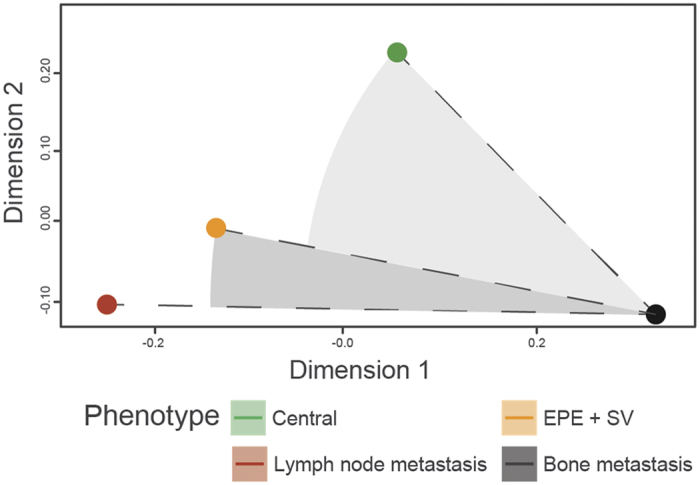
Multi-dimensional scaling plot of the centroids of the increase/decrease copy number profiles representative of the transitions between the four histo-pathological categories (i.e., from diploid-to-central tumour, central-to-EPE, EPE-to-lymph node metastasis, and central/EPE-to-bony metastasis). Representative copy number profiles for distant metastases were taken from patients 001, 177, 299, 498; representative copy number profiles for the other histo-pathological categories were taken from patients 167, 179 and 421.

Finally, we sought a measure to determine the genome-wide relatedness between lymph, bone, EPE and central tumours that utilised the tumour phylogenies. CNA profiles belonging to the same histo-pathological category were averaged, obtaining four centroid profiles for each category. We then calculated the Euclidean distance between each applying a bootstrap leave-one-out method that produced seven distinct distance matrices, which showed consistent results to the predicted solution in Fig. 4.

The analysis of the Euclidean distance across copy-number profiles (Table S2) surprisingly indicated that the profiles of bone metastases were generally more related with the primary prostate tumour populations than with the lymph node metastases. The large separation between lymph and bone metastases using this measure suggests they are distinct at a genomic level.

## Discussion

In this study we explored the genomic relationships between distinct metastatic populations in a small number of advanced prostate cancer patients. Inspection of the evolutionary hierarchies that we derived for patients with lymph node metastases, 167, 179 and 421, indicated that these metastases most likely originated from tumour cell populations that had already extended beyond the prostate capsule and which therefore exhibited an invasive phenotype, rather than from more centrally located and less invasive tumour cell populations, such as in the case for distant bone metastases. This suggests that lymph node tumours may not act as a conduit point for further tumour cell dissemination, at least not to more distant osseous or visceral sites. These observations are also consistent with the model of metastatic progression that emerges from a similar study of advanced prostate cancer patients^10^. In this study, the evolutionary hierarchies that include lymph node metastases (i.e., patients A17, A22, A24, A31 and A32) consistently show that lymph node metastasis is not a common ancestor for distant bone metastases, but rather represents an independent evolutionary route emerging from central tumours. Furthermore, a recent study for brain metastases^29^ identified regional lymph node metastases as highly divergent from the metastases located in the brain; supporting the hypothesis of diverse evolutionary histories of local or distant metastases.

These results, though not by any means definitive due to the limited sample size, do seem to accord with the observed differences in clinical behaviour of different anatomical sites of metastasis in advanced prostate cancer patients. Prior clinical studies documenting metastatic status and oncological outcomes in prostate cancer patients have lent weight to the hypothesis that lymph node metastasis is a distinctive biological entity compared to metastases presenting at bony or visceral sites. For instance it has been demonstrated that there is a clear differential in the survival status of patients with node only disease even after primary treatment, compared to those with more distant metastases^30^. More recent studies are now also corroborating that the survival rates of patients presenting with metastatic disease is significantly linked to both the site and number of metastatic deposits. Individuals from a large contemporary cohort of prostate cancer patients presenting with either exclusive visceral metastases or additionally with bone involvement, had poorer survival than those with bone only metastases^31^. The number of metastatic sites involved also impacted on survival with patients having multiple metastatic sites having a significantly shorter survival than patients with oligometastatic disease^31^. Interestingly, patients with a single visceral metastasis experienced a higher mortality rate than patients with bony metastatic disease. Patients however who exhibited only lymph node involvement had the best mortality outcomes of any of the metastatic patients and a significantly better overall and cancer specific survival than patients with visceral metastases. It is noteworthy that all the patients from this study had stage IV disease at presentation and furthermore the node only disease patients were only diagnosed using the relatively insensitive imaging modalities of CT and MRI and hence it is likely that their true nodal disease burden was in fact underestimated. It is tempting to speculate that the clear differences in oncological outcomes of these patients based on the location of the metastatic deposits reflects underlying biological differences in the nature of the tumour cell clones that give rise to the distinctive patterns of metastatic disease.

There has been very little genetic investigation of the potential underlying differences in metastatic lesions based on site of presentation in prostate cancer; however work in the field of breast cancer has shed some light on this issue. Data from preclinical modelling of breast cancer metastasis indicates that distinct populations of tumour cells in the primary organ drive spread to lymph nodes and that these cells possess distinctive epigenetic profiles that predispose them to disseminate and proliferate in the lymph nodes rather than bony sites^32^. In breast cancer, patients with positive lymph nodes have a clearly increased risk of disease recurrence following lymphadenectomy surgery^33^. Additionally, lymph node status is the single most important prognostic feature in breast cancer and in this regard the disease differs from prostate cancer. Recently, it has been demonstrated that differential methylation of the *PAX6* gene was found to be linked to lymph node metastasis in breast cancer, with positive nodes having significantly lower levels of *PAX6* methylation than paired and matching primary tumour tissues from the same cohort of 25 patients^34^. These results further strengthen the hypothesis that specific tumour cell clones with distinctive epigenetic changes might disseminate and colonize specifically the lymph node niche in cancer patients.

A number of key questions are yet to be resolved concerning the mechanisms of distinctive tumour cell dissemination. For example, do cells with these distinctive genetic changes arise stochastically in unselected populations localized within the primary tumour or do metastasis specific changes occur first in the prostate tumour cells and then once the cells have lodged in specific anatomical locations are they then further selected for changes that predispose them to proliferate and colonize distinctive metastatic niches? Certainly our results suggest that the tumour cell populations that seed to either lymphatic or bony sites arise from distinct tumour locations in the prostate and may therefore be indicative of the biological characteristics of tumour cell population of origin as a whole.

Resolving the issue of whether positive lymph nodes can commonly act as a conduit for more distant bony or visceral tumour dissemination is not just of academic interest but has clear clinical implications. This is particularly so in the setting of the burgeoning uptake in the new and highly sensitive imaging modality of PSMA PET^35^,^36^,^37^,^38^,^39^,^40^. With the increasing use of this screening technology an increasing number of node only as well as node plus osseous and/or visceral sites metastatic patients are likely to be identified.

This study represents a foundation for a further investigation of the behavioural patterns of prostate cancer metastases at distinct sites. Such improved knowledge will allow greater metastatic control as well as the employment of therapies that target selectively high risk lesions. This will result in decreased systemic treatment toxicity compared to current systemic therapies that assume all metastatic lesions are equivalent.

## Methods

All experimental protocols and all methods described here were approved and carried out in accordance with the guidelines of the Epworth Hospital Human Ethics committee.

### Patient selection

Following informed consent from all patients and institutional ethics approval from the Epworth Hospital Human Ethics committee, suitable prostate cancer specimens were identified from three patients with N1 disease following radical prostatectomy and lymph node dissection (i.e., 167, 179 and 421) from our institutional database^41^. As a comparison arm, we included in the study a further four patients with biopsy confirmed bony metastatic disease following radical prostatectomy. All patients prospectively consented to the use of their tissues for genomics studies in accordance with the core bioethical elements for genomics projects. Clinico-pathological details of these three patients are presented in Table S1. All three patients presented with large, advanced stage, primary tumours. No patient had more than one lymph node histologically confirmed to contain malignancy. The prostate specimens were fixed in formalin after collection. Glands were sliced at 4–5 mm intervals perpendicular to the urethra, with the apex and base slices additionally sectioned in the parasagittal plane at 4 mm intervals. A 5 mm slice was then subsequently made from each section, stained with haematoxylin and eosin (H&E) and all tumour borders marked by pen. These slides were then scanned and a tumour map constructed depicting graphically the topography and extent of prostate cancer in each gland as well as any features of prognostic relevance, such as seminal vesical involvement^42^. All pathological examinations of specimens were undertaken prospectively and shared within a group of four urological pathologists with regular internal audit practices for consistency.

### Tissue processing

We identified regions of interest based on topographical depiction of tumour location, extent and foci in relation to the rest of the prostate gland; and targeted areas of tumour that defined high-risk disease, including seminal vesicle invasion and extraprostatic extension, as well as adjacent central portions of tumour and other foci of lower grade cancer. We also included positive surgical margins as these areas could represent the advancing front of the tumour. For lymph nodes, we targeted tumour at the capsule of the lymph node at the marginal sinus, considering that this region is prone to early deposits of metastatic prostate cancer.

We then cut 5 μm reference slide from archival blocks of formalin-fixed paraffin-embedded (FFPE) containing regions of interest.

The slide was H&E stained and boundaries of tumour foci were marked with felt-tip pen on the cover slips. A further six slides of 10 μm thickness were cut from each block for macrodissection. The unstained slides were deparaffinised in xylene, washed with a decreasing ethanol series and nuclease-free water, and H&E stained with a rapid protocol. The slides remained immersed in ethanol until ready for dissection.

We used three to six slides per region of interest depending on the likely DNA yield; and macrodissected each region of interest using 27.5 G hypodermic needles and number 15 scalpel blades at 8–35× magnification (Leica EZ4 HD stereomicroscope).

### DNA Extraction and SNP Chip

Genomic DNA was extracted using the QIAamp DNA FFPE Tissue Kit (Qiagen, Maryland, Ca) according to manufacturer’s instructions. Briefly, we placed the macrodissected tissue in 180 μl ATL buffer with 20 μl proteinase K, and performed RNAse digestion and genomic DNA purification by column chromatography with elution in 100 μl nuclease-free water. We restored 200 ng of genomic DNA per sample using the Infinium HD FFPE Restore Protocol (Illumina) and Zymo Research DNA Clean & Concentrator (Zymo) as required. For each sample, all 8 μl of restored DNA was used as input for the Infinium HD FFPE Assay (Illumina). We linearly amplified the DNA across the whole genome and fragmented it enzymatically. The resulting product was hybridised to the Illumina HumanOmniExpress-FFPE-12 v1.0 BeadChip and incubated at 48 °C for 16–24 hrs. Imaging was performed using the Illumina iScan system and intensity values derived for each bead type. LogR Ratios and B allele frequencies (BAF) were calculated from the intensity data using GenomeStudio v2010.3 with Genotyping module 1.8.4 software and the HumanOmniExpress-12v1 G FFPE manifest cluster file. Overall, we achieved high SNP call rates with a median of 98.35% (range 92.47% to 99.72%). The median standard deviation of the LogR Ratio values was 55%. The goodness of fit of the predicted aberrant fractions was always above 0.76 with a mean of 0.91.

### Phylogenetic analyses

For the prediction of gene copy number (CN) profiles, tumour cellularity and ploidy of the patients 167, 179, 421 (for which sample collection, processing and molecular analysis is described above), and for patients 001, 177, 299, 298 (for which sample collection, processing and molecular analysis has been described previously^11^) we employed ASCAT^43^ with default settings. For each of those patients, with the exception of patient 177 (for which no central tumour was available), we inferred the tumour evolutionary hierarchy from the copy number profiles (Fig. S1). First, we fragmented the genomic breakpoints (defined as the start and end of the genomic regions affected by CNA) of all samples to obtain comparable CNA profiles, and then selected CNAs originated from large genomic autosomal fragments (>500.000 base pairs) for further analyses. The program MEDICC^18^ was used (with default settings) to infer the evolutionary hierarchy from the CNA profiles; we then annotated each branch (i.e., monophyletic clade) of the resulting evolutionary hierarchy with a confidence score (referred as subsampling confidence) using an iterative chromosome subsampling approach. For this approach, the steps described above were iterated 30 times using random subsamples (without replacement) of 15 autosomes at the time; the frequency at which a selected monophyletic clade in the source evolutionary hierarchy appeared in the sampling simulations indicated the confidence of that clade. The branches of the source evolutionary hierarchy with a low confidence score (<0.5; i.e., majority rule) were then collapsed to their most proximal predicted ancestral cell population (referred from now on as the ancestor). The majority of the branches derived from the predicted evolutionary hierarchies (Fig. 2) had a subsampling confidence higher than 0.5 (i.e., appeared in greater than 50% of the sampling simulations). The only exceptions were the branching nodes L, H and v from patient 167 that had a subsampling confidence of 0.46 and were collapsed to node iv, and for the branching nodes B and D from patient 299 that had a subsampling confidence of 0.43 and were collapsed to node i. The shoulder metastasis from patient 299 was placed in the evolutionary hierarchy based on additional genomic information from Hong *et al*.^11^. Such an approach was employed because this particular sample was analysed with a higher density SNP array, compared with the other samples in the same evolutionary hierarchy, introducing a clear bias that prevented high confidence predictions, based solely on SNP array information.

### Comparative analyses of copy number profiles

For identifying the potential driver CNAs across transitions between histo-pathological categories, we selected for each evolutionary hierarchy the representative ancestral population for each category; with the criteria that it should originate all (or the majority) of sampled clones belonging to such category. For each transition between histo-pathological categories, we obtained the increase/decrease CNA profile subtracting two consecutive representatives (e.g., central subtracted from extraprostatic extension). Then, we consistently fragmented the resulting increase/decrease CNA profiles to allow direct comparison and tested each genomic fragment for differences across histo-pathological categories using analysis of variance (ANOVA; function aov from R platform). All changes with a false-discovery corrected (method Benjamini, Hochberg, and Yekutieli^44^) p-value <0.25 are reported in Table 2.

### Validation

We independently validated the CNA profiles inferred using ASCAT from SNP array data using the copy numbers predicted from Multiplex Ligation-dependent Probe Amplification (MLPA) technology^45^ on 58 genomic regions spanning 39 genes (kit ID P294-B1) over the samples 167C, 167D, 167E, 167G, 167H, 167J, 167L, 179C, 179D, 179E, 179G, 179L, 421C, 421E, 421F, 421G, 421H and 421L. MLPA was performed as per vendor’s protocol (MRC-Holland, Amsterdam, The Netherlands). We normalized the raw MLPA intensities for probe length using the function mlpaNorm (using the method “slope correction”) from the R package MLPAstats^46^, and calculated the ratio between the normalized MLPA intensity values for genes in tumour samples and benign, control samples (i.e., 167B, 179B, 421A). Then, from those ratios, we inferred the gene CNs using thresholds adjusted for tumour ploidy (Fig. S3) according to the formula *ratio -* (*ploidy-2*)/*2*. Each branch of the evolutionary hierarchies was considered validated if a significant proportion of genes with a variation in CN was validated; a gene was considered validated if the copy number obtained using MLPA and SNParray was the same. Evolutionary hierarchies for patients 001, 177, 299 and 498 were indirectly validated comparing their topologies with the topologies of the evolutionary hierarchies obtained with somatic point mutations and structural variation analysis identified from whole genome sequencing^11^.

### Data and computational algorithms

The raw data of the SNP arrays for patients 167, 179, 421 can be retrieved at ega-archive.org with the code EGAS00001001801. The informatics code used for the analyses in this work can be retrieved at github.com/stemangiola/PC-lymph-node-mets-evolution.

## Additional Information

**How to cite this article**: Mangiola, S. *et al*. Comparing nodal versus bony metastatic spread using tumour phylogenies. *Sci. Rep.* **6**, 33918; doi: 10.1038/srep33918 (2016).

## Supplementary Material

Supplementary Information

## Figures and Tables

**Table 1:** Validation using MLPA.

Patient	Node	descendants	Probes validated (%)	P-value	Adjusted p-value
167	i	ii; iii	76.3	2.863e-11	1.7178e-10
167	iii	iv; D	73.7	2.551e-10	6.1224e-10
167	iv	v; L/H	70.0	7.343e-09	1.4686e-08
179	i	ii; vi	73.7	2.551e-10	6.1224e-10
179	ii	iii; v	73.7	2.551e-10	6.1224e-10
179	iii	iv; G	87.5	<2.2e-16	2.64e-15
421	i	ii; B	60.7	3.104e-05	3.7248e-05
421	ii	iii; D	60.7	3.104e-05	3.7248e-05
421	iii	iv; C	60.7	3.104e-05	3.7248e-05
412	iv	v; H	61.3	7.819e-06	1.3404e-05
421	v	iv; F	57.1	2.159e-04	0.0002159
421	vi	vii; L	59.0	6.764e-05	7.378909e-05

Significance calculated with binomial test with p = 0.33. P-values were corrected for multiple test with the Benjamini, Hochberg, and Yekutieli method^44^.

**Table 2:** Genes within the top 10 regions for differential gain/loss copy number aberration.

Chromosome	Locus	Gene	Copy number increase/decrease (mean)				P-value	Adjusted p-value
			Central	Extraprostatic	Lymph node	Bone		
16	16q24.2	FBXO31	−0.33	0.25	−1.67	0.01	0.0006	0.0312
11	11p15.5	MUC2	0	−0.5	−0.67	0.69	0.0033	0.08013
11	11p15.5	MUC6	0	−0.5	−0.67	0.69	0.0033	0.08013
11	11p15.5	KRTAP5-5	0	−0.5	−0.67	0.69	0.0033	0.08013
1	1p36.33	CFAP74	0	−0.67	−1	0.41	0.0157	0.11452
16	16p13.3	CREBBP	0	0.25	−1	0.5	0.0175	0.11452
8	8q24.3	GML	0.8	−0.67	−1	0.54	0.0207	0.11452
8	8q24.3	MAFA	0.8	−0.67	−1	0.54	0.0207	0.11452
4	4q24	TET2	0.67	−0.5	0.33	0.22	0.0385	0.18741
4	4q24	NPNT	0.67	−0.5	0.33	0.22	0.0385	0.18741
19	19q13.4	BRSK1	0.17	0.25	−0.67	0.51	0.0497	0.18741
8	8q24	RAD21	1.4	−0.67	0.5	1.65	0.0604	0.21143
5	5p15.2	SEMA5A	0	0.5	−0.33	1.01	0.0701	0.21206
8	8q23.1	ANGPT1	1.6	−0.67	0.5	1.65	0.0741	0.21206
8	8q22	RUNX1T1	1.6	−0.67	0.5	1.65	0.0741	0.21206
8	8q22.2	COX6C	1.6	−0.67	0.5	1.65	0.0741	0.21206
8	8q21	NBN	1.6	−0.67	0.5	1.65	0.0741	0.21206
8	8q22.3	RIMS2	1.6	−0.67	0.5	1.65	0.0741	0.21206
8	8q22.2	CPQ	1.6	−0.67	0.5	1.65	0.0741	0.21206
8	8q21	WWP1	1.6	−0.67	0.5	1.65	0.0741	0.21206
8	8q21	HEY1	1.6	−0.67	0.5	1.65	0.0741	0.21206
8	8q22.2-q23	PABPC1	1.6	−0.67	0.5	1.65	0.0741	0.21206
8	8q22	UBR5	1.6	−0.67	0.5	1.65	0.0741	0.21206
8	8q21.13	PAG1	1.6	−0.67	0.5	1.65	0.0741	0.21206
8	8q23	DCSTAMP	1.6	−0.67	0.5	1.65	0.0741	0.21206
8	8q23	PKHD1L1	1.6	−0.67	0.5	1.65	0.0741	0.21206
8	8q23.3	CSMD3	1.6	−0.67	0.5	1.65	0.0741	0.21206
8	8q21.3	DCAF4L2	1.6	−0.67	0.5	1.65	0.0741	0.21206
8	8q21.3	SLC7A13	1.6	−0.67	0.5	1.65	0.0741	0.21206
8	8q22.3	SNX31	1.6	−0.67	0.5	1.65	0.0741	0.21206
8	8q23.1	RSPO2	1.6	−0.67	0.5	1.65	0.0741	0.21206
