# Supplementary material for: Comparing nodal versus bony metastatic spread using tumour phylogenies
Source: Sci Rep. 2016 Sep 22;6:33918. doi: 10.1038/srep33918 (PMC5031992; doi:10.1038/srep33918)
Supplement: Supplementary Information [file srep33918-s1.doc]

# SUPPLEMENTARY MATERIAL

For the article: Comparing nodal versus bony metastatic spread using tumour phylogenies

# Stefano Mangiola^1,5,*^, Matthew KH Hong^1^, Marek Cmero^1^, Natalie Kurganovs^1^, Andrew Ryan^4^, Anthony J Costello^1, 2^, Niall M Corcoran^1,2^, Geoff Macintyre^3,5,*^, Christopher M Hovens^1,2,*^

# Department of Surgery, Division of Urology, Royal Melbourne Hospital and University of Melbourne, Parkville 3050 Victoria Australia

# 2. The Epworth Prostate Centre, Epworth Hospital, Richmond 3121, Victoria, Australia

# 3. Cancer Research UK Cambridge Institute, University of Cambridge, Cambridge CB2 0RE, UK

4. TissuPath Specialist Pathology, Mount Waverley 3149, Victoria, Australia.

5. Centre for Neural Engineering, 203 Bouverie St, Carlton 3053, Victoria, Australia.

*Corresponding authors:

S. Mangiola, Department of Surgery, Royal Melbourne Hospital, University of Melbourne, 5^th^ Floor Clinical Sciences Bldg, Royal Parade, Parkville, 3050 VIC, Australia. E-mail: smangiola@unimelb.edu.au

G. Macintyre, Cancer Research UK Cambridge Institute, University of Cambridge, Li Ka Shing Centre, Robinson Way, Cambridge CB2 0RE

gm513@cam.ac.uk

**Fig. S1** Pipeline for data production and processing.


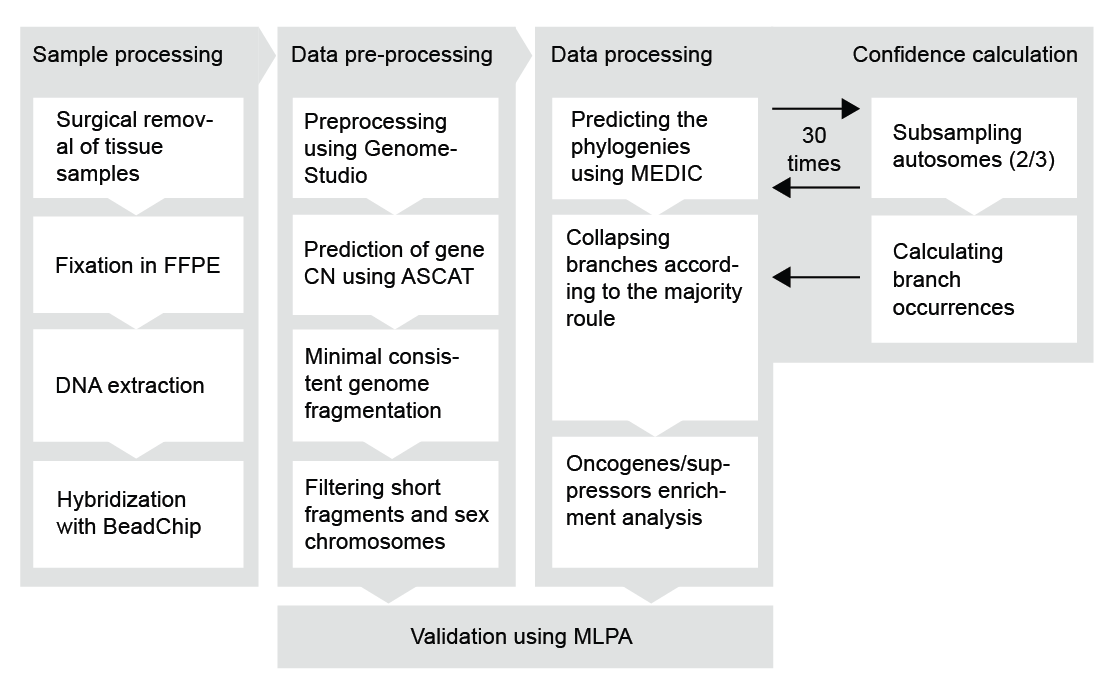


**Table S1.** Sample information

| **sample type** | **Patient** | **Purity** | **Ploidy** | **Sample ID** |
| --- | --- | --- | --- | --- |
| Central | 167 | 0.63 | 2.11 | C |
| Central | 167 | 0.6 | 3.07 | D |
| Extraprostatic | 167 | 0.56 | 2.06 | E |
| Extraprostatic | 167 | 0.62 | 3.17 | G |
| Extraprostatic | 167 | 0.6 | 2.92 | H |
| Extraprostatic | 167 | 0.59 | 2.96 | J |
| Lymph node metastasis | 167 | 0.66 | 3.03 | L |
| Central | 179 | 0.55 | 2.18 | C |
| Central | 179 | 0.62 | 2.19 | D |
| Extraprostatic | 179 | 0.44 | 2.27 | E |
| Extraprostatic | 179 | 0.48 | 2.01 | F1 |
| Extraprostatic | 179 | 0.52 | 2.1 | F2 |
| Central | 179 | 0.75 | 2.07 | G |
| Lymph node metastasis | 179 | 0.46 | 1.88 | L |
| Extraprostatic | 177 | 0.68 | 1.93 | A |
| Distant metastasis | 177 | 0.23 | 1.87 | C |
| Distant metastasis | 177 | 0.21 | 1.92 | I |
| Central | 421 | 0.67 | 2.17 | B |
| Central | 421 | 0.42 | 1.96 | C |
| Central | 421 | 0.56 | 2.25 | D |
| Extraprostatic | 421 | 0.5 | 1.92 | E |
| Extraprostatic | 421 | 0.48 | 2.3 | F |
| Extraprostatic | 421 | 0.52 | 1.77 | G |
| Central | 421 | 0.43 | 1.89 | H |
| Lymph node metastasis | 421 | 0.53 | 1.98 | L |
| Distant metastasis | 001 | 0.64 | 2.16 | Cancer |
| Central | 001 | 0.44 | 2.27 | A |
| Distant metastasis | 001 | 0.49 | 2.26 | B |
| Distant metastasis | 001 | 0.46 | 2.22 | G |
| Central | 498 | 0.4 | 3.92 | C1 |
| Central | 498 | 0.39 | 1.75 | C2 |
| Central | 498 | 0.39 | 1.86 | D |
| Distant metastasis | 498 | 0.82 | 3.03 | A |
| Distant metastasis | 498 | 0.8 | 2.74 | recurrence |
| Distant metastasis | 498 | 0.22 | 3.3 | C |
| Distant metastasis | 498 | 0.53 | 2.8 | FT |
| Central | 299 | 0.64 | 2.01 | B |
| Central | 299 | 0.84 | 1.98 | C |
| Extraprostatic | 299 | 0.84 | 2.02 | D |
| Central | 299 | 0.56 | 1.86 | E |
| Extraprostatic | 299 | 0.86 | 1.94 | F |
| Central | 299 | 0.46 | 1.81 | G |
| Central | 299 | 0.52 | 1.94 | H |
| Central | 299 | 0.36 | 3.81 | J |
| Extraprostatic | 299 | 0.52 | 1.93 | K |
| Central | 299 | 0.35 | 1.89 | M |
| Central | 299 | 0.4 | 1.88 | N |
| Distant metastasis | 299 | 0.75 | 2.13 | met |

**Table S2.** Euclidean distance between the copy number profiles of distant metastases from patients 001, 177, 299 and 498, and the samples for the other histo-pathological categories from patients 167, 179 and 421. Each row represents a bootstrap run (leave-one-out). In all bootstrap runs the distant metastasis has a more similar profile to the extra prostatic tumour populations than to the lymph node metastasis.

| **Central** | **Extraprostatic** | **Lymph node metastasis** |
| --- | --- | --- |
| 15.25942 | 10.30713 | 12.66807 |
| 18.91113 | 10.55563 | 13.145 |
| 14.13868 | 10.18786 | 12.13361 |
| 19.76523 | 12.31086 | 13.19241 |
| 16.2067 | 11.86406 | 14.29403 |
| 10.33433 | 9.870683 | 13.84044 |
| 15.36157 | 10.74516 | 12.97997 |
